# Therapeutic efficacy of artemether–lumefantrine for the treatment of uncomplicated *Plasmodium falciparum* malaria in Enfranze, north-west Ethiopia

**DOI:** 10.1186/s12936-015-0775-3

**Published:** 2015-06-24

**Authors:** Gebeyaw Getnet, Abebe Alemu Fola, Agersew Alemu, Sisay Getie, Hans-Peter Fuehrer, Harald Noedl

**Affiliations:** Department of Medical Parasitology, School of Biomedical and Laboratory Sciences, College of Medicine and Health Sciences, University of Gondar, Gondar, Ethiopia; School of Medicine, College of Health Sciences and Medicine, Wolaita Sodo University, Wolaita, Ethiopia; The Walter and Eliza Hall Institute of Medical Research, Melbourne, Australia; Department of Pathobiology, Institute of Parasitology, University of Veterinary Medicine Vienna, Vienna, Austria; Institute of Specific Prophylaxis and Tropical Medicine, Medical University of Vienna, Vienna, Austria

**Keywords:** Efficacy, *Plasmodium falciparum*, Artemether–lumefantrine, Enfranze

## Abstract

**Background:**

*Plasmodium falciparum* accounts for approximately 60% of malaria cases in Ethiopia and artemether–lumefantrine has been used as a first-line treatment for uncomplicated *P. falciparum* malaria since 2004. The aim of this study was to assess the therapeutic efficacy of artemether–lumefantrine (AL) for the treatment of uncomplicated *P. falciparum* malaria in north-western Ethiopia.

**Methods:**

A 28-day one-arm, prospective evaluation of the clinical and parasitological response to the first-line treatment for uncomplicated *P. falciparum* malaria was conducted in Enfranze Health Centre in accordance with the 2009 WHO efficacy study guidelines. Patients were treated with a 3-day course of AL and clinical and parasitological parameters were monitored over a 28-day follow-up. All data from recruited patients were imported into an electronic data base and Kaplan–Meier survival analysis was used for analysing primary [early treatment failures (ETF), late clinical failure (LCF), late parasitological failures (LPF), and adequate clinical and parasitological response (ACPR)] and secondary (PCT, GCT and FCT) outcomes.

**Results:**

Eighty patients were enrolled and all of them completed the 28-day follow-up period. The PCR-corrected cure rate was 95.0% (95% CI 87.0–98.4%) and there were two ETF, one LCF and three LPF. Two of the LPF were classified as re infections by PCR. Seventy three point seven five percent, 91.25 and 95% of patients had cleared their parasitaemia by days 1, 2, and 3, respectively, and 75, 91.25 and 96.25% of patients had cleared their fever by days 1, 2, and 3. All patients completely cleared their gametocytes by day 7.

**Conclusion:**

The relatively high cure rate, low proportion of patients still positive on day 3 as well as parasite clearance times in this study would indicate no imminent threat of artemisinin resistance development in the region. However, the threat of spreading or de novo development of artemisinin resistance warrants regular monitoring of drug efficacy throughout the region.

## Background

Malaria is a disease caused by protozoan parasites of the genus *Plasmodium* and transmitted by female *Anopheles* mosquitoes. *Plasmodium falciparum* is by far the most important specie, responsible for nearly all severe malaria cases [1, 2]. About 198 million cases of malaria occurred globally in 2013 and the disease led to 584,000 deaths. The burden is heaviest in the WHO African Region, where an estimated 90% of all malaria deaths occur, and in children <5 years of age, who account for 78% of all deaths [3].

Malaria is the leading communicable disease in Ethiopia and an estimated 68% of the population of Ethiopia lives in areas at risk of malaria [4]. In the country, *P. falciparum* and *Plasmodium vivax* are the main species accounting for roughly 60 and 40% of malaria cases, respectively [5, 6]. *Plasmodium falciparum* causes severe malaria with a case fatality rate of about 10% in hospitalized adults and up to 33% in children less than 12 years old in Ethiopia [7].

Early diagnosis and prompt treatment is one of the main strategies in malaria prevention and control and it is also the key to reducing morbidity and preventing mortality [8]. However, efforts towards controlling malaria are greatly challenged by the increasing spread of anti-malarial drug resistance [3]. Use of ineffective anti-malarial drugs contributes to the difficulties in reducing malaria morbidity and mortality, leads to the spread of malaria to new areas, re-emergence of malaria in areas where the disease had been eliminated and it has also played an important role in the occurrence and severity of epidemics [9]. Anti-malarial drug resistance is observed in *P. falciparum* malaria, but has also been identified in *P. vivax* [10, 11].

In Ethiopia, high level resistance to chloroquine (CQ) in 1998 necessitated a change to sulfadoxine–pyrimethamine (SP) as first-line anti-malarial drug. However, high treatment failure rates with SP of up to 72% were reported in some areas which have led to increasing acceptance of using a combination of two or more drugs in an attempt to reduce malaria transmission and resistance development. Consequently, artemether–lumefantrine (AL) was adopted in 2004, which currently is being used as the first-line drug for the treatment of uncomplicated *P. falciparum* malaria [8]. A base-line study in 2004 showed that AL was a highly efficacious drug with a treatment success of 99.1% and with few reports of adverse effects [12, 13].

The World Health Organization (WHO) recommends artemisinin-based combinations, such as AL, as first-line treatment forum complicated *P. falciparum* malaria in all endemic countries [14]. However, resistance of *P. falciparum* to artemisinin has been confirmed in western Cambodia and Thailand [15]. This resistance has spread from the Thai–Cambodia border to the Greater Mekong region, up to the border of Myanmar and India [16–19]. This resistance has the potential to spread to or develop de novo in other parts of the world [20] and drug efficacy may slowly deteriorate over time. The WHO recommends regular monitoring of drug efficacy for the first line anti-malarial drugs at defined sentinel sites at least once every 2 years in order to detect changes in their therapeutic efficacy [14]. The main focus of this study was to assess the therapeutic efficacy of AL in the treatment of uncomplicated *P. falciparum* malaria in north-western Ethiopia.

## Methods

### Study area

The study was conducted at Enfranze Health Centre. Enfranze is a sub-district, located in North Gondar administrative zone, Amhara Region, 675 km north of Addis Ababa and 60 km from Gondar town and at an elevation of 1,500 m above sea level. This area is malaria-endemic with a total population of about 45,686 (municipality report) and the majority of the population depends on subsistence farming.

### Study design and period

The design was a one-arm, prospective evaluation of the clinical and parasitological response to directly observed treatment for uncomplicated *P. falciparum* malaria conducted between January and May 2013.

### Study subjects

The study subjects were recruited among febrile patients attending Enfranze Health Centre using inclusion criteria as defined in the WHO guidelines for assessing the therapeutic efficacy of anti-malarial drugs against *P. falciparum* malaria [21].

### Inclusion criteria and exclusion criteria

The following inclusion criteria were used for the study: mono-infection with *P. falciparum*, above 6 months of age, a parasitaemia of 1,000—100,000/μl, weight >5 kg, presence of axillary temperature (≥37.5°C) and no use of anti-malarial drugs 2 weeks prior to enrollment into the study. Patients with danger signs of severe and complicated malaria according to WHO criteria (including severe anaemia defined as haemoglobin <5 g/dl), history of allergic reactions to the study drug AL, mixed infection with another *Plasmodium* species, concomitant presence of febrile conditions with the potential to confound study outcome (e.g. acute respiratory infection, severe diarrhoea or other known underlying chronic or severe diseases (e.g. cardiac, renal or hepatic diseases, HIV/AIDS), severe malnutrition (defined as a child whose growth standard is below −3 z-score, has symmetrical oedema involving at least the feet or has a mid-upper arm circumference <110 mm for 6 month–18 years of age children and has a mid-upper arm circumference <170 mm, BMI <16 with or has a mid-upper arm circumference <180 mm with recent weight loss or underlying chronic illness for adults), as well as pregnant and lactating women, were not included in the study.

### Sample size and sampling technique

The sample size was determined using a single population proportion formula according to the WHO guidelines: assuming a maximum of 25% clinical failures, 10% precision, and a confidence level of 95% with up to 10% losses to follow up a sample size of 80 was calculated [21].

### Data collection procedures

A rapid screening procedure was used in an outpatient setting to identify patients who meet enrolment criteria. The typical screening data set included age, sex, temperature, body weight, pregnancy test, initial blood slide examination and haemoglobin. All patients meeting the basic enrolment criteria during the screening procedure were evaluated in greater depth by a member of the study team. Physical examination was performed at baseline (day 0 before dosing) and on days 1, 2, 3, 7, 14, 21 and 28. Body weight was determined on day 0 using a weight scale. The screening weight was used to calculate the dose (number of tablets) to be administered. Axillary temperature was measured at baseline (day 0 before dosing) and on days 1, 2, 3, 7, 14, 21 and 28. Female patients of child-bearing age (12–49 years) were asked to provide a urine sample for pregnancy testing before enrolment in the study and if sexually active were provided with condoms for the duration of the study.

### Sample collection and processing

Finger-prick blood samples were collected from consenting patients for malaria parasite identification and haemoglobin level measurement. Patients that satisfied the criteria were enrolled into the study and followed up on days 1, 2, 3, 7, 14, 21, and 28 where finger-prick samples were taken for microscopic glass slides. Another drop of blood was collected on Whatman 903^®^ filter paper on day 0 during enrollment and in case of recurrent parasitaemia. The filter paper was air dried and stored in a self-sealing plastic bag with desiccators for further molecular analysis.

### Microscopic diagnosis of malaria parasites

Thick and thin blood smears were prepared and stained with 10% Giemsa (pH 7.4) for 10 min and read by two senior microscopists. Blood films were taken at least eight times for each patient during the study period (day 0, 1, 2, 3, 7, 14, 21 and 28) and during any unscheduled visit. A blood film was considered negative when no parasites were seen after examining 100 high power fields on the thick film. Parasites were counted on thick films relative to 200 leukocytes by two microscopists blinded to each other’s results. Blood smears with discordant results (differences between the two microscopists in species diagnosis, or differences in parasite density of >50%) were re-examined by a third, independent microscopist, and parasite density was calculated by averaging the two closest counts.

### Genotyping of malaria parasites

In order to differentiate a recrudescence from a newly acquired infection, blood spots were collected from all patients at day 0 (before drug intake) and in case of LPF on Whatman filter paper and sent to Medical University of Vienna for genotyping of merozoite surface protein 1 (MSP1), merozoite surface protein 2 (MSP2) and glutamate-rich protein (GLURP). To exclude mixed infections or infections with other human malaria parasites the samples were analysed with nested PCR for species classification as reported previously [22, 23]. Afterwards *P. falciparum* monoinfections were genotyped. Gene loci—*glurp*, *msp1* and *msp2*—of these samples were compared by PCR as described previously [24].

### Haematological assessment

Finger-prick blood samples were used to measure haemoglobin. Due to limited resources the actual haemoglobin concentration should be measured by hemocue. However, this study assessed the haematocrit value only. In healthy persons, the haematocrit (expressed as a percentage) is roughly three times the haemoglobin concentration (expressed in grams per decilitre). This ratio is maintained in normocytic anaemia, but in most of the tropical forms of chronic anaemia the ratio is 3.3:1.

### Treatment and follow-up of patients

All eligible patients were treated with AL (Coartem^®^) (Novartis Pharmaceutical Corporation, Suffern, New York, USA for Novartis Pharma AG, Basel, Switzerland, and Bach No. F-2832) twice daily on days 0, 1, and 2. Study participants were advised to take the study drug with milk to improve absorption. Study medication was administered based on weight; the first and each morning dose were directly observed by the study staff [25]. The evening doses were given to the patient/guardian for self-administration in the presence of health extension workers. Patients were followed for 30 min post-treatment and if vomiting occurred, a second full dose was administered. If repeated vomiting occurred, patients were withdrawn from the study. Patients were asked to return to the health centre on days 1, 2, 3, 7, 14, 21, and 28 or whenever they did not feel well. Patients withdrawn or with complications were referred to the health centre for proper treatment. Patients experiencing a reemergence of *P. falciparum* parasitaemia were treated with quinine.

### In vivo analysis and classification response

Patients were classified as early treatment failure (ETF), late clinical failure (LCF), and late parasitological failure (LPF, adequate clinical and parasitological response (ACPR) as per WHO definition [21].

### Data analysis

After checking for completeness all data were imported into Excel and Kaplan–Meier survival analysis was used for analysing primary (ETF, LCF, LPF, and ACPR) and secondary (PCT, FCT, GCT) outcomes, Cox regression was used to identify predictor variables of secondary outcomes. P values <0.05 were considered statistically significant.

### Ethical consideration

The study protocol was reviewed and approved by the Ethical Review Committee of the School of Biomedical and Laboratory Sciences, College of Medicine and Health Sciences, University of Gondar. Written informed consent was obtained from all study participants or their legal representatives after being translated and read in the vernacular language.

## Results

### Study participants

A total of 80 patients (46 males and 34 females) were enrolled. Baseline demographics are presented in Table 1. Not a single participant was lost to follow-up. Headache, dizziness, cough, anorexia, and diarrhoea were the most commonly reported adverse events in 26, 15, 25, 12 and 8% of the patients, respectively. Across age groups participants had similar median parasite densities, proportion of gametocyte carriage and mean temperature (Table 1).

**Table 1:** Demographics of study participants at Enfranze Health Centre

	≤5 years old	**>**5 years old	Total
	N = 16	N = 64	N = 80
Mean age (SD)	3.61 (0.75)	23.4 (14.9)	19.4 (15.5)
Male no (%)	10 (62.5)	36 (56.3)	46 (57.5)
Mean weight kg (SD)	12.9 (1.64)	40.6 (15.6)	35 (17.9)
Mean temp °C (SD)	38.5 (0.72)	38.2 (0.5)	38.3 (0.56)
Median parasite load/μl (IQR)	7,360 (6,190–8,720)	7,675 (6,360–8,742)	7,898 (6,360–8,742)
Mean Hgb g/dl (SD)	11.6 (1.2)	12.4 (1.8)	12.3 (1.8)
Gametocyte carriage no (%)	3 (18.75%)	5 (7.8%)	8 (10%)

### Cure rate of AL against uncomplicated *Plasmodium falciparum* malaria

The PCR-corrected cure rate by Kaplan–Meier analysis was 95.0% (95% CI 87.0–98.4%). Two participants developed ETF on days 2 and 3, respectively. One participant had a LCF on day 14 and three participants developed LPF on days 21 and 28, respectively, out of which two were classified as re-infections based on molecular analysis of paired samples. The remaining 74 patients completed the follow-up without recurrence of parasitaemia (Table 2).

**Table 2:** PCR uncorrected and corrected 28 days cure rate of AL as well as early and late treatment failures

Outcome	≤5 years (no = 16)	>5 years (no = 64)	Total (no = 80)
ETF	0	2 (3.1%)	2 (2.5%)
LCF	1 (6.25%)	0	1 (1.25%)
LPF	1 (6.25%)	2 (3.1%)	3 (3.75%)
ACPR	14 (87.5%)	60 (93.75%)	74 (92.5%)
Cure rate (uncorrected)	14 (87.5%)	60 (93.75%)	74 (92.5%)
Cure rate (PCR-corrected)	14 (87.5%)	62 (96.9%)	76 (95%)

*ETF* early treatment failure, *LCF* late clinical failure, *LPF* late parasitological failure, *ACPR* adequate clinical and parasitological response.

### Effect of treatment with AL on parasite, fever, and gametocyte clearance rates

Seventy three point seven five percent, 91.25 and 96.2% of patients cleared their parasites by days 1, 2, and 3, respectively. The overall mean parasite clearance time was 33.6 ± 2.16 h. Seventy five percent, 91.25, and 97.5% of patients cleared their fever on day 1, 2, and 3, respectively. The overall mean fever clearance time was 33 ± 2.020 h. On day 1, all three patients ≤5 years old with gametocytes had cleared their gametocytes where as in the five patients >5 years old gametocytes progressively decreased and completely cleared by day 7 (Table 3).

**Table 3:** Proportion of patients with parasite, fever and gametocyte clearance on days 1, 2, and 3, respectively

Out come	≤5 years old	>5 years old	Total
Parasites cleared			
D1 no (%)	12 (75)	47 (73.4)	21 (73.75)
D2 no (%)	14 (87.5)	59 (92.2)	73 (91.25)
D3 no (%)	15 (93.75)	61 (95.2)	76 (94.9)
Fever cleared			
D1 no (%)	10 (62.5)	50 (78.1)	60 (75)
D2 no (%)	13 (81.25)	60 (93.75)	73 (91.25)
D3 no (%)	14 (87.5)	63 (98.4)	77 (96.2)
Gametocytes cleared			
D1 no (%)	0	2 (40)	2 (25)
D2 no (%)	0	3 (60)	3 (37.5)
D3 no (%)	0	3 (60)	3 (37.5)

*D1* day 1, *D2* day 2, *D3* day 3.

### Parasite densities and temperature at baseline, parasite, fever and gametocyte clearance

Based on parasite density at baseline participants had similar mean fever (P = 0.97) and gametocyte (P = 0.798) clearance times. There was statistically significant association between parasite density at baseline and mean parasite clearance time (P = 0.002) (Table 4).

**Table 4:** Parasite densities at base line versus mean parasite, fever and gametocyte clearance time among patients treated with AL

Base line parasite density (no)	Mean PCT	*P* value	Mean FCT	P value	Mean GCT	P value
≤6,360 (22)	24		30.5		24	
6,361–7,675 (18)	24		32		36	
7,676–8,743 (20)	26.4		33.6		60	
≥8,744 (20)	60		36		60	
Total (80)	33.6	0.002	33	0.97	45	0.798

The statistical tool used to calculate p value is Cox regression.

*PCT* parasite clearance time, *FCT* fever clearance time, *GCT* gametocyte clearance time.

Based on axillary temperatures at baseline participants had similar mean parasite (P = 0.760), fever (P = 0.329) and mean gametocyte (P = 0.498) clearance time (Table 5).

**Table 5:** Temperature at base line versus mean parasite, fever and gametocyte clearance time among patients treated with AL

Temperature at base line (°C) (no)	Mean PCT	P value	Mean FCT	P value	Mean GCT	P value
≤37.8 (22)	30.5		24		–	
37.9–38 (20)	37.2		30		32	
38.1–38.8 (22)	39.5		38		48	
≥38.9 (16)	39		42		60	
Total (80)	33.6	0.760	33	0.329	45	0.498

*PCT* parasite clearance time, *FCT* fever clearance time, *GCT* gametocyte clearance time.

## Discussion

This study suggests that with a cure rate of around 95% AL remains an efficacious treatment for uncomplicated *P. falciparum* malaria in the region. These results are consistent with studies reported from neighboring Kenya, 96% [25] and Burkina Faso, 96.6% [26] or Togo, 93% [27] and slightly lower than previously reported from e.g. India, 99.9% [28], Senegal, 100% [29], Congo, 100% [30], Tanzania, 100% [31], southern Ethiopia, 99.4% [32] and south-western Ethiopia, 97.5% [33]. Most of these results are well within the confidence intervals of this study and minor differences may be attributable to regional variations in the duration of deployment of AL before the studies were conducted, the age distribution of participants, levels of prevalence (and, therefore, immunity), as well as drug administration practices (e.g. in terms of administering with/without food).

Unlike previous reports from south-western Ethiopia [33], participants in this study ≤5 years old had a slightly, but not significantly (P = 0.18) lower cure rate than those >5 years. At least in part this may be attributable to the fact that the study explicitly did not interfere with the way children are fed and to a low-milk/fat diet typically given to children in the study region, which may have caused poorer absorption and cure rates in small children.

Unlike most previous studies, in this study there were two ETF [32–34]. The two ETF were one male participant 18 years old with parasite densities of 11,000/µl of blood and one male participant 17 years old with parasite densities 10,000/µl. Both had initial parasite densities above the mean and were classified as ETF based on development of severe malaria day 2 in the presence of parasitaemia and parasitaemia on day 3 with axillary temperature ≥37.5°C.

All except seven participants had cleared parasitaemia by day 2. However, four patients (5%; 95% CI 2.0–12.2) were still parasitaemic on day 3 (72 h) after initiation of treatment. All of these patients had initial parasite counts above average and one of them (a 5-year-old female participant with 18,000 parasites/µl) later developed a LCF. This is considered to be well below the threshold indicating potentially emerging resistance and is comparable to previous findings in southern Ethiopia [32] and Burkina Faso [26] and considerably lower than the 21.9% parasitaemic patients on day 3 reported from a trial conducted in western Cambodia as early as 2007 [15]. With only 33.6 h in spite of comparable initial parasite densities the parasite clearance times were also considerable shorter than those seen in Southeast Asia [35].

Clinical improvement was swift and fever clearance was similarly rapid in most of the participants with only three participants (3.75%; 95% CI 1.28–10.45) remaining febrile up to day 3. Fever clearance largely depends on inclusion criteria (e.g. febrile vs. only history of fever) and is, therefore, difficult to compare across study sites but seemed similar to fever clearance reported in previous studies across Africa [26, 30, 36, 37]. All eight participants with gametocytaemia on enrollment had cleared their gametocytes by day 7. Although this may seem faster than some previous reports from Africa, the relatively small number does not allow for conclusions of the potential influence of AL treatment on malaria transmission.

## Conclusion

The relatively high cure rate, low proportion of patients still positive on day 3 as well as parasite clearance times in this study would indicate no imminent threat of artemisinin resistance development in the region. Artemether–lumefantrine remains highly efficacious in the treatment of uncomplicated falciparum malaria in small as well as older children and adults. However, the threat of spreading or de novo development of artemisinin resistance warrants regular monitoring of drug efficacy throughout the region.
